# Healing‐assessment tools for perineal and cesarean section wounds in postpartum women: A scoping review

**DOI:** 10.1111/aogs.70089

**Published:** 2025-11-11

**Authors:** Rebecca Man, Jack Le Vance, Yasmine Popa, Danielle Wilson, Sue Tohill, John Maltby, Victoria Hodgetts Morton, R. Katie Morris, the CHAPTER group, Christine MacArthur, Laura Magill, Laura Jones, Sara Webb, Sarah Hillman, Nicola J. Adderley, Alice Sitch, Olalekan Lee Aiyegbusi, Marian Knight, Krishnarajah Nirantharakumar, Jane Whitehurst

**Affiliations:** ^1^ Department of Applied Health Sciences, School of Health Sciences, College of Medicine and Health University of Birmingham Birmingham UK; ^2^ Birmingham Women's Hospital Birmingham UK; ^3^ College of Medicine and Health University of Birmingham Birmingham UK; ^4^ Department of Population Health Sciences University of Leicester Leicester UK

**Keywords:** birth‐related wound, childbirth, episiotomy, perineal tear, perineal trauma, perineal wounds, vaginal birth, wound‐assessment tool, wound infection

## Abstract

**Introduction:**

Approximately 85% of women who undergo vaginal birth sustain childbirth‐related perineal trauma. Worldwide, 21% of women give birth by cesarean section. These wounds therefore affect the vast majority of women after birth; however, there is a lack of validated tools to accurately identify women with abnormal wound healing in the postpartum period. Consequently, in clinical settings, validated wound‐assessment tools are not generally used to assess wound healing in this population. We performed a scoping review to identify and characterize wound‐assessment tools that have been used to determine the healing of childbirth‐related wounds in existing research (to include women who experience perineal trauma or a cesarean section).

**Material and Methods:**

Medline, EMBASE, CINAHL, and Google Scholar were searched from inception to April 2024. Studies were included where wound‐assessment tools were used to assess wound healing, after women had sustained either childbirth‐related perineal trauma or a cesarean section, as an outcome of a primary research article. For studies that assessed wound healing in women with perineal trauma, this included all types of childbirth‐related perineal trauma, sustained at spontaneous or assisted vaginal birth. Studies were eligible for inclusion where the wound‐assessment tool was used at any time‐point in the postpartum period.

**Results:**

There were 95 studies eligible for inclusion; 72 of which utilized wound‐assessment tools for the assessment of healing after women sustained childbirth‐related perineal trauma and 23 for women with cesarean section wounds. The REEDA tool (redness, oedema, ecchymosis, discharge, approximation) was used in 91 of the 95 studies, with the remainder using alternative wound‐assessment tools, including the use of the ASEPSIS tool (additional treatment, serous discharge, erythema, purulent exudate, separation of deep tissues, isolation of bacteria, and length of inpatient stay).

**Conclusions:**

There are limited wound‐assessment tools to determine healing after women sustain childbirth‐related wounds. The REEDA tool is the most commonly used in research settings. There is a clear need for the development of a clinically robust and inclusive wound‐ assessment tools, which comprehensively reflect the postpartum healing process among diverse populations.

## Graphical abstract

Limited assessment tools exist to determine healing after childbirth‐related wounds. The REEDA tool is commonly used in research studies; however, it is not widely used clinically. To facilitate accurate assessment among diverse populations, we recommend the development of a new tool. Created in BioRender. Man, R. (2025) https://BioRender.com/nvg88u4.

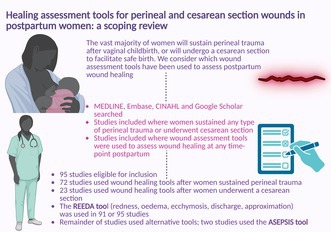

**Key message**

Limited assessment tools exist to determine healing after childbirth‐related wounds. The REEDA tool is commonly used in research studies. To facilitate accurate assessment among diverse populations, we recommend the development of a new tool.

## 1 INTRODUCTION

The majority of women who give birth will sustain a resulting wound, either from vaginal birth (episiotomy or a spontaneous perineal tear) or from a cesarean section.^1^, ^2^ Approximately 85% of women who undergo vaginal birth will sustain perineal trauma—90% in first births and 69% in subsequent births.^1^ Worldwide estimates currently suggest that 21% of women undergo a cesarean section for birth.^2^ Postnatal infection rates vary significantly, with perineal wound infections estimated to affect between 0.1% and 23.6% of women, and surgical site infections after cesarean section estimated to affect approximately 6% of women. These estimates vary depending on definitions and assessment methods.^3^, ^4^

Despite these wounds occurring in the vast majority of women who give birth and wound complications occurring frequently, postnatal care and access to appropriate healthcare professionals remain limited.^5^ If complications are not identified and treated efficiently and effectively, then women can face significant physical consequences, such as pain, the need for further surgical intervention, sepsis, and fistula formation. The impacts of these complications can be wide‐reaching, with detrimental effects on mental health, the ability to bond with their new baby, relationships with other family members, and the ability to undertake physical activity or activities of daily living.^6^, ^7^, ^8^, ^9^, ^10^

The 2022 World Health Organisation recommendations on Maternal and Newborn Care for a Positive Postnatal Experience, emphasize the importance of wound assessments at each postnatal contact.^11^ Despite these clear global recommendations, women highlight feeling that postnatal wound assessment is often neglected, particularly after they have sustained perineal trauma.^9^, ^12^ There is currently no routine wound‐assessment tool in widespread clinical use to determine healing at any time point in the postnatal period after women sustain childbirth‐related wounds (perineal trauma or a cesarean section). As such, clinicians express uncertainty in the management of childbirth‐related wounds in the postnatal period, which may lead to delays in women receiving the care they require.^13^ The development of a robust and comprehensive wound‐assessment tool to guide clinicians in detecting abnormal wound healing would help mitigate these existing barriers to care. As part of considering the development of a new wound‐assessment tool, it is imperative to consider which tools are currently in existence.

To our knowledge, there is no existing systematically performed review that identifies wound‐assessment tools that have been used to assess wound healing after women sustain childbirth‐related perineal trauma or undergo cesarean section. While there are previous reviews investigating which wound‐assessment tools are available across different types of wounds,^14^, ^15^ it is crucial that childbirth‐related wounds are considered to be a separate entity. Childbirth‐related wounds occur via a distinct mechanism of injury and in a specific population. Women also have unique requirements placed on them in the postpartum period, notably the care of their newborn. It is therefore important that we undertake a focused review specific to this population.

Here, we perform a scoping review to identify and characterize wound‐assessment tools that have been used to determine the healing of childbirth‐related wounds in existing research (to include women who experience perineal trauma or a cesarean section). This review was undertaken with the aim of answering the research question: which wound‐assessment tools have been used in existing research studies to determine wound healing, where women have undergone childbirth‐related perineal trauma or a cesarean section and what are the characteristics of those tools?

## 2 MATERIAL AND METHODS

### 2.1 Study design

This study forms a scoping review of wound‐assessment tools used in primary research studies to assess healing after women sustain childbirth‐related perineal trauma or undergo cesarean section. Scoping review methodology was utilized, as our research question pertained to which wound‐assessment tools existed for this population, as opposed to a more focused question, for example, investigating effectiveness with an aim to inform clinical decision‐making.^16^ Additionally, our aims were not to perform a synthesis of quantitative data statistically or to combine qualitative data, but rather to identify what currently exists—the latter lends itself well to scoping review methodology while the former is more inherent to systematic reviews.^17^

Although PROSPERO does not register scoping reviews per se, this review was conducted as part of a broader registered protocol for the overall program (CRD42023458738). The Joanna Briggs Institute framework on scoping reviews was used to guide the review process.^18^ This scoping review was reported as per the PRISMA guidance for scoping reviews (PRISMA‐ScR).^19^

### 2.2 Inclusion and exclusion criteria

#### 2.2.1 Inclusion criteria

Primary research studies were included where they used a wound‐assessment tool to determine wound healing after women sustained childbirth‐related perineal trauma or underwent a cesarean section. Although the original protocol focused on perineal trauma, we expanded the scope to include cesarean section wounds based on stakeholder input, to ensure broader applicability to postnatal care.

Studies including women with all types of perineal trauma, sustained at spontaneous or assisted vaginal birth were included. Studies including women whose wound healing was assessed across all ethnic and cultural groups, and all age groups were eligible for inclusion. There were no constraints placed on geographical location. We included studies where the wound‐assessment tool was applied at any time point within the postnatal period to assess wound healing. This decision was made to ensure that no potentially relevant wound‐assessment tools were excluded on the basis of primary study authors applying the tool at a particular time point in order to meet the broad aims of this scoping review. A wound‐assessment tool was identified if the study utilized a range of symptoms or signs to determine an overall score, which established how well the wound was healing, or if a previously validated tool was used, as identified by the authors.

#### 2.2.2 Exclusion criteria

Publications that were not primary research, such as systematic reviews and editorials or where a wound‐assessment tool was being developed and tested, rather than used to determine healing as a study outcome, were excluded. We chose to exclude studies that described tool development, as we aimed to determine which wound‐assessment tools were currently in existence, with subsequent real‐life application to women who had sustained childbirth‐related wounds. If we were to include hypothetical tools that had not been applied to the relevant population, then the resulting inventory of individual tool items may not present potentially applicable components. Studies that used tools purely for assessment of the presence or absence of wound infection, rather than a global healing scale, or only to determine scar cosmesis, were excluded. Where studies used tools to determine the degree of perineal tear immediately after perineal trauma and not to determine healing, they were excluded. Non‐English language studies were excluded due to limited translation resources.

Appendix S2 categorizes the aforementioned inclusion and exclusion criteria into population, concept, context (PCC), as is aligned with best practices for performing scoping reviews.

### 2.3 Search strategy

Systematic literature searches were undertaken in the Embase, Medline and CINAHL databases in addition to the Google Scholar search engine (first 200 results from Google Scholar) from inception to April 2024. Embase and Medline were utilized as they are the two primary biomedical literature databases.^20^ By combining both databases, even in the absence of any other sources, it is estimated that approximately 90% of relevant references will be found where the topic of interest is relevant to human health.^21^ The decision to include CINAHL was made, because this database has a focus on nursing and allied health subjects including midwifery; therefore, it is pertinent when retrieving articles about wound healing. Google Scholar was utilized, as research indicates that inclusion of the first 200 results returned from Google Scholar (ordered by default in terms of relevance) contributes to optimal citation retrieval.^22^ Google Scholar, used as an adjunct to database searching, is a powerful engine for finding relevant gray literature on a subject, which may not otherwise be retrieved.^23^ A combination of medical subject headings (MeSH) and text words were used, with the search terms adapted to the specifics of each database. MeSH/database specific indexing terms included “Wounds and Injuries,” “Perineum,” “Injury,” “Episiotomy,” “Cesarean Section,” “Wound Healing,” “Wound Assessment,” “Healing.” A full list of search terms can be found in Supporting Information: Appendix S1.

### 2.4 Study selection and data extraction

Two reviewers independently reviewed titles and abstracts (R.M, J.L.V) with any discrepancies resolved by consensus of authors. Each study progressing to full‐text review was screened for eligibility by two authors (R.M., Y.P., D.W., or S.T.) with discrepancies resolved by consensus. This process was repeated for data extraction, with each study independently extracted by two reviewers (R.M., J.L.V., Y.P., D.W., or S.T.) and conflicts resolved by consensus. Covidence software was used to organize the screening process. For eligible studies, data was charted onto a pre‐designed electronic spreadsheet, after piloting. Data extracted from each study included author, year of publication, location of study, aims, type of wound assessed for healing, timing of wound evaluation, tool type and characteristics. After piloting the data collection spreadsheet, criteria related specifically to how the REEDA (redness, oedema, ecchymosis, discharge, approximation) tool had been applied in the primary study were added. These included whether authors had calculated a total score from 15, whether the total score was then attributed to a categorical measure of healing and whether authors had specified how each parameter was scored.

Characteristics of wound‐assessment tools recorded included the parameters which comprised the tool and the scoring system. Where wound‐assessment tools were not named as a recognized tool but where the components of the tool were stated and matched that of the established tool, then it was recorded as the recognized tool. Where authors did not explicitly state the components used in their tool within their methods or results, then we did not make assumptions about the parameters used.

## 3 RESULTS

### 3.1 Study selection and study characteristics

**FIGURE 1:**
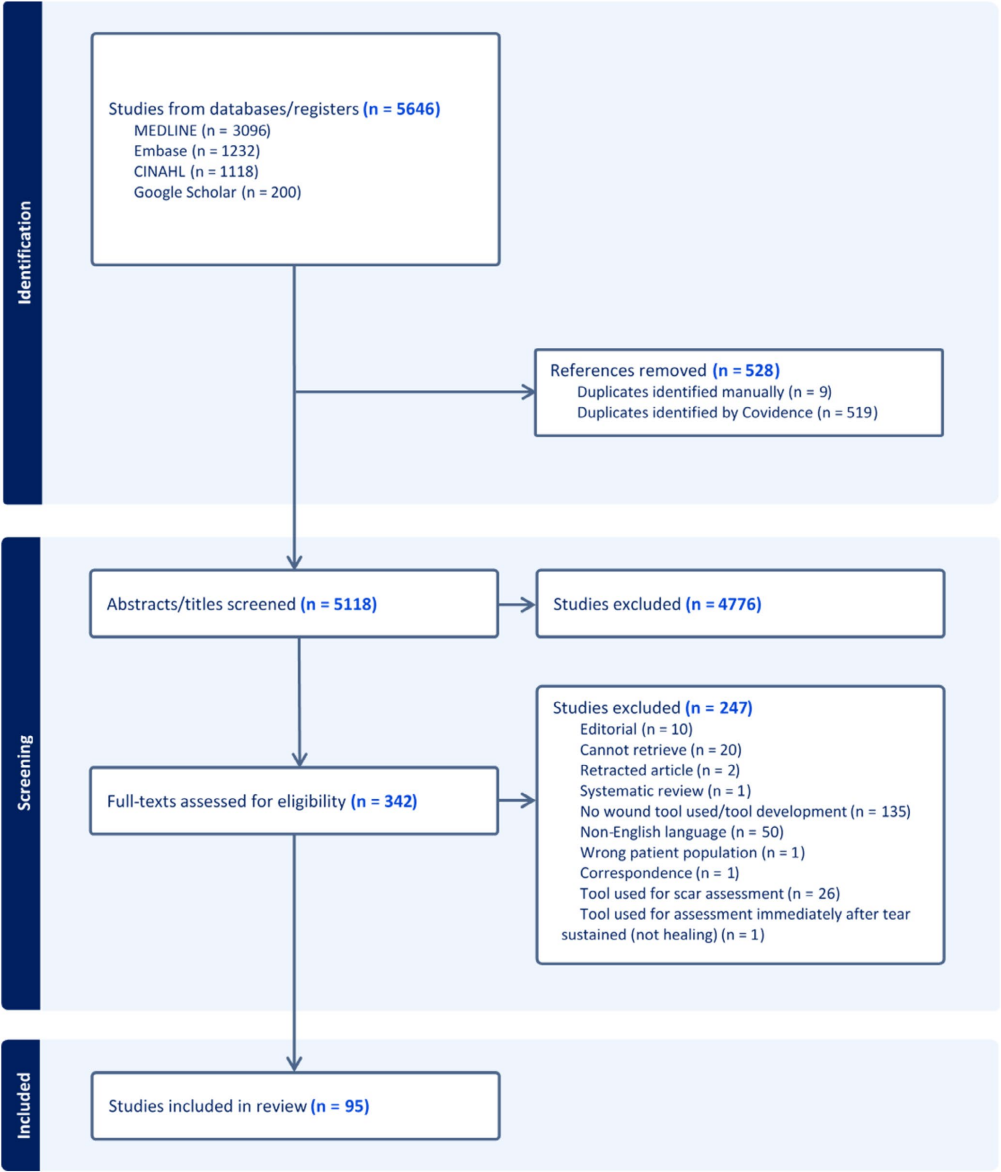
PRISMA flow diagram showing study selection.

There were 95 studies eligible for inclusion, from a total of 5118 initial titles/abstracts screened (Figure 1: PRISMA flow diagram).^24^, ^25^, ^26^, ^27^, ^28^, ^29^, ^30^, ^31^, ^32^, ^33^, ^34^, ^35^, ^36^, ^37^, ^38^, ^39^, ^40^, ^41^, ^42^, ^43^, ^44^, ^45^, ^46^, ^47^, ^48^, ^49^, ^50^, ^51^, ^52^, ^53^, ^54^, ^55^, ^56^, ^57^, ^58^, ^59^, ^60^, ^61^, ^62^, ^63^, ^64^, ^65^, ^66^, ^67^, ^68^, ^69^, ^70^, ^71^, ^72^, ^73^, ^74^, ^75^, ^76^, ^77^, ^78^, ^79^, ^80^, ^81^, ^82^, ^83^, ^84^, ^85^, ^86^, ^87^, ^88^, ^89^, ^90^, ^91^, ^92^, ^93^, ^94^, ^95^, ^96^, ^97^, ^98^, ^99^, ^100^, ^101^, ^102^, ^103^, ^104^, ^105^, ^106^, ^107^, ^108^, ^109^, ^110^, ^111^, ^112^, ^113^, ^114^, ^115^, ^116^, ^117^, ^118^ Studies were conducted in 18 countries across the world, with 45 studies conducted in Iran (47%), 19 in India (20%) and the remainder in Brazil (6%), Turkey (4%), Egypt (3%), the United Kingdom (3%), China (2%), Indonesia (2%), the United States (2%), Singapore (1%), Pakistan (1%), Philippines (1%), Denmark (1%), Spain (1%), Israel (1%), Romania (1%), France (1%), and Italy (1%) (Supporting Information Table S1: study characteristics). The number of women included in each study ranged from 19 to 1314. There were 72 studies (76%) that investigated the use of a wound‐assessment tool after women sustained childbirth‐related perineal trauma (episiotomy *n* = 59, episiotomy/second‐degree tear *n* = 4, episiotomy/first/second‐degree tear *n* = 4, first‐degree tear *n* = 2, second‐degree tear *n* = 1, first/second‐degree tear *n* = 1, all types of perineal trauma *n* = 1) and 23 (24%) after women underwent cesarean section.

The majority of included studies were randomized controlled trials (*n* = 72), in addition to quasi‐experimental studies (*n* = 18), cohort studies (*n* = 3), case series (*n* = 1), and cross‐sectional studies (*n* = 1). Studies investigating an intervention included a wide range of potential treatments or management options, including different surgical materials or methods for repair, wound dressings, hot/cold therapy, topical medications, and an array of alternative therapies.

### 3.2 Wound‐assessment tools used and their characteristics

#### 3.2.1 REEDA score

The most commonly used wound‐assessment tool was the REEDA (redness, oedema, ecchymosis, discharge, approximation) tool, used in 91 of 95 studies (96%). Of the 72 studies utilizing a wound‐assessment tool to determine healing after childbirth‐related perineal trauma, 70 (97%) used the REEDA scale. Of the 23 studies investigating healing after cesarean section, 21 studies (91%) utilized the REEDA scale.

Included studies gave different levels of detail about how they had used the REEDA scale and applied the tool in various ways. In 17 of 91 studies (19%), it was explicitly documented how each parameter within the REEDA scale was scored. The majority of studies assigned scores of zero to three for each parameter (a lower score reflecting a better wound condition). In 76 of 91 studies (84%), the total REEDA score was calculated from 15, to give a global healing score. There were 17 of 91 studies (19%) that had attributed ranges of total REEDA scores to different degrees of overall healing (e.g., 0 = improvement, 1–5 = moderate improvement, 6–10 = weak recovery, 11–15 = no recovery). In studies within this review, the REEDA tool was used to measure wound healing from the day of birth, to postpartum week eight.

#### 3.2.2 ASEPSIS score

The ASEPSIS (additional treatment, serous discharge, erythema, purulent exudate, separation of deep tissues, isolation of bacteria and length of inpatient stay) tool was used as a measure of wound healing in two studies, both of which were investigating the healing of cesarean section wounds.

Majid et al. utilized the ASEPSIS score to compare the healing of post‐cesarean wounds using different dressings.^73^ The authors measured the ASEPSIS score at postpartum days 5, 7, 9, and 10. Majid et al. assigned scores as per the following: antibiotic use 10 points, drainage of pus 5 points, debridement of wound (with general anesthesia) 5 points, serous discharge 0–5 scale, erythema 0–5 scale, purulent exudate 0–10 scale, separation of deep tissues 0–10 scale, isolation of bacteria 10 points, stay in hospital prolonged for over 14 days 5 points. Kilic et al. used the ASEPSIS tool (modified version) to determine wound healing after cesarean section, in women assigned to have their wound dressings removed at 24 h versus 48 h postpartum.^66^ Women's wounds were assessed using the ASEPSIS tool at postpartum weeks 1 and 6. Authors interpreted a total score of zero as complete healing and over zero as incomplete healing, with higher scores equating to increasingly severe healing disruption.

#### 3.2.3 Other wound‐assessment tools

Two studies used alternative wound‐scoring tools. Greer et al. used their own wound‐ assessment tool to determine the healing of episiotomy wounds in their trial comparing different topical healing foams.^49^ Their assessment criteria and scoring system are demonstrated in Table 1. The sum of all the parameters was then calculated to give a global healing score at the different time points. David et al. used a wound‐healing tool to assess the effects of a nonsteroidal anti‐inflammatory drug on the healing of episiotomy wounds.^36^ Here, oedema, redness, and pain were assessed on a scale of zero to three (0 = none, 3 = severe) with a total then calculated, giving an overall healing score. A total score of zero to three was considered to be good healing, 4–6 moderate healing and 7 or over, poor. The tool was used at postpartum days 1, 3, and 5.

**TABLE 1:** Alternative wound‐scoring tool by Greer et al.^49^

Assessment criteria for days 1, 3 and 5		
Oedema	Marked	4
	Moderate	3
	Minimal	2
	Nil	1
Erythema	Marked	4
	Moderate	3
	Minimal	2
	Nil	1
Pain	Severe	4
	Moderate	3
	Mild	2
	None	1
Impairment of mobility secondary to episiotomy pain	Marked decrease	4
	Moderate decrease	3
	Minimal decrease	2
	No change	1
Analgesia required for episiotomy pain	Four times a day or more	4
	Three times a day	3
	Two times a day	2
	Once a day or less	1

Additional criteria assessed only on day 5		
Subjective overall response to treatment	None	4
	Poor	3
	Fair	2
	Good	1
Healing	Breakdown	4
	Poor	3
	Satisfactory	2
	Good	1

#### 3.2.4 Overall tool components

The most commonly assessed symptoms in wound healing were visual indicators, such as redness, erythema, oedema, discharge, and approximation, with each of these being recorded in 88–89% of studies (Table 2). Ecchymosis was reported in 82% of studies. Other clinical indicators, such as the need for additional treatment (e.g., antibiotics or debridement), bacterial isolation, and extended inpatient stays, were reported in 2% of studies. Women's experience of pain and their use of analgesia were incorporated as components of wound‐assessment tools in 2% and 1% of studies, respectively.

**TABLE 2:** Frequency of symptoms/signs or clinical measures recorded in included studies.

Component of tool	Number (percentage) of included studies which recorded using the component as part of their wound‐assessment tool, *n* = 95
Redness or erythema	85 (89%)
Oedema	84 (88%)
Discharge	84 (88%)
Approximation	84 (88%)
Ecchymosis	82 (86%)
Additional treatment (drainage/antibiotics/debridement)	2 (2%)
Isolation of bacteria	2 (2%)
Stay in hospital over 14 days	2 (2%)
Pain	2 (2%)
Impairment of mobility	1 (1%)
Analgesia	1 (1%)

## 4 DISCUSSION

The most commonly used wound‐assessment tool to assess healing after childbirth‐related wounds was the REEDA tool. How well the tool was applied within the studies included in this review was variable, with the majority of studies not explicitly defining how scoring within a parameter was assigned. The REEDA tool was developed in the 1970s in order to assess the healing of postpartum perineal wounds.^119^, ^120^ A score of 0–3 is established for each parameter of the tool (0 representing normal wound healing and 3 representing severely disrupted wound healing) with a total of 15 calculated from the sum of each parameter. Table 3 demonstrates the scoring criteria in the original REEDA scale.^119^ The REEDA tool was originally validated for use in women with episiotomy and perineal trauma wounds; however, a number of included studies within this scoping review used the REEDA to determine wound healing after women underwent a cesarean section.^119^, ^121^

**TABLE 3:** Components and scoring of the REEDA (redness, oedema, ecchymosis, discharge, approximation) scale.^119^

	Redness	Oedema	Ecchymosis	Discharge	Approximation
0	None	None	None	None	None
1	Within 0.25 cm of the incision bilaterally	Less than 1 cm from incision	Within 0.25 cm bilaterally or 0.5 cm unilaterally	Serious	Skin separation 3 mm or less
2	Within 0.5 cm of incision bilaterally	Between 1 and 2 cm from incision	Between 0.25 cm and 1 cm bilaterally or between 0.5 and 2 cm unilaterally	Serosanguinous	Skin and subcutaneous fat separation
3	Beyond 0.5 cm of incision bilaterally	Greater than 2 cm from incision	Greater than 1 cm bilaterally or 2 cm unilaterally	Bloody, purulent	Skin, subcutaneous fat and fascial layer separation
Score					
Total					

In addition to the REEDA tool, the ASEPSIS tool was used in two studies to determine healing after women underwent cesarean section The ASEPSIS score was originally developed as a wound‐scoring method in a trial investigating antibiotic therapy after cardiac surgery and has points assigned to each parameter (Table 4).^122^ A total is then calculated, with 0–10 representing satisfactory healing, 11–20 disturbed healing, 21–30 minor wound infection, 31–40 moderate wound infection and over 40 severe wound infection. Certain parameters were given scores only within the first 7 days postoperatively with the remainder assigned points up to 2 months postoperatively in the original study (details in Table 4).^122^ Points for the tool parameters scored within a range are assigned based on the percentage of the total wound affected by the particular sign (Table 5).

**TABLE 4:** Components and scoring of the ASEPSIS tool (as per the original study by Wilson et al.).^122^

Component	Points
Additional treatment	
Antibiotics	10
Drainage of pus under local anesthesia	5
Debridement of wound (general anesthesia)	10
Serous discharge^a^	0–5
Erythema^a^	0–5
Purulent exudate^a^	0–10
Separation of deep tissues^a^	0–10
Isolation of bacteria	10
Stay as inpatient prolonged over 14 days	5

^a^ Scored only on the first 7 days postoperatively.

**TABLE 5:** Scoring system for each parameter within the ASEPSIS tool (as per the original study by Wilson et al.).^122^

Wound characteristic	Proportion of wound affected (%)					
	0	<20	20–39	40–59	60–79	>80
Serous exudate	0	1	2	3	4	5
Erythema	0	1	2	3	4	5
Purulent exudate	0	2	4	6	8	10
Separation of deep tissues	0	2	4	6	8	10

None of the wound‐assessment tools reviewed, particularly in terms of their parameters, offer full coverage of critical factors needed for a comprehensive evaluation of wound healing among women with childbirth‐related wounds. Notably, none of these tools, including REEDA, ASEPSIS, Greer et al., and David et al., adequately address skin tone, a significant omission that raises concerns about their universal applicability. Skin color plays an essential role in visual assessments of wounds—particularly relevant to indicators like erythema which may appear as various changes in skin color, not just limited to redness.^123^

To our knowledge, there has been no other scoping review performed on this subject, meaning that we fill an important gap in the evidence base. By identifying the frequency and variety of individual components within these tools, we provide a foundation for future development of a new, validated wound‐assessment tool. The symptoms and signs identified through this review, with refinement, can serve as potential items in the inventory for future tools, having been previously recognized as important in the literature.

There are also limitations to our study. This review included only studies published in English, which may have resulted in the exclusion of relevant wound‐assessment tools used in studies published in other languages. Additionally, we restricted our inclusion criteria to tools used as outcome measures in primary research, excluding studies focused on the development or validation of new tools. This may mean that some tools under development, or those not yet employed as primary research outcomes, were omitted.

Consideration must also be given to the potential for publication bias. Smaller unfunded studies, trials with negative findings, or studies without the input of senior researchers, unfortunately may be less likely to be published. Therefore, in the absence of publication, these studies would be omitted from this scoping review. Furthermore, as the identification of relevant wound‐assessment tools was a finding of our review (and not known a priori), we acknowledge that we may not have identified every existing study using each wound‐assessment tool, as the existing tool acronyms were by default not included in the database search terms. This is not thought to be a limitation of pragmatic relevance, as our aim was primarily to identify the existing tools—there would be little benefit to finding an increased number of studies, all of which used the same tool. Additionally, we have not performed critical appraisal for studies included in this review. Critical appraisal is not inherent to scoping review methodology; however, we felt it was important to consider whether performing this step would strengthen this work. Given that the primary aim of our work was to determine which wound‐assessment tools had been used in existing studies, we felt that presenting a risk of bias assessment for each included study may not provide additional constructive information.

Based on our findings, we propose areas where we recommend that further research on the subject should focus. First, both the REEDA and ASEPSIS tools were developed without sufficient stakeholder engagement, including input from women who had experienced childbirth‐related wounds. Involving key stakeholders in future tool development will enhance clinical relevance and ensure the tools are pragmatic and sensitive to women's needs. Additionally, we recommend broadening the parameters considered for inclusion in any newly developed wound‐assessment tool. Current tools rely heavily on visual symptoms, with a paucity of more patient‐centered measures. Future tools should consider measures like pain and mobility, to offer a holistic assessment of recovery. There is also a substantial need to increase representation in tool development studies, to ensure the tool is designed and tested among women with a range of different skin tones. Through this scoping review, we provide, to our knowledge, the first systematically performed review investigating wound‐assessment tools for women who have sustained childbirth‐related wounds. This provides the necessary building blocks for future work in this area.

## 5 CONCLUSION

This scoping review reveals that the REEDA tool is the most widely used for assessing the healing of childbirth‐related wounds, although it does not account for women with different skin tones. The REEDA tool also relies purely on visual indicators of healing with no patient‐reported components. To address these limitations, the development of future wound‐assessment tools should involve key stakeholders from the outset, crucially including women who have experienced childbirth‐related wounds. The tool should be developed and tested with women from a diverse range of ethnic backgrounds and among women who have sustained the specific type of childbirth‐related wound for which the tool is proposed to be applied. To our knowledge this is the first scoping review that investigates wound‐assessment tools for women with childbirth‐related wounds and as such this review is key to underpinning future research on this subject.

## AUTHOR CONTRIBUTIONS

Rebecca Man: conceptualization, data curation, methodology, writing–original draft, writing–review and editing. Jack Le Vance: data curation. Yasmine Popa: data curation. Danielle Wilson: data curation. Sue Tohill: data curation. John Maltby: writing–review and editing. Victoria Hodgetts Morton: supervision, writing–review and editing. R Katie Morris: supervision, writing–review and editing.

## CHAPTER GROUP

Christine MacArthur, Department of Applied Health Sciences, School of Health Sciences, College of Medicine and Health, University of Birmingham, Edgbaston, Birmingham, UK. Laura Magill, Department of Applied Health Sciences, School of Health Sciences, College of Medicine and Health, University of Birmingham, Edgbaston, Birmingham, UK. Laura Jones, Department of Applied Health Sciences, School of Health Sciences, College of Medicine and Health, University of Birmingham, Edgbaston, Birmingham, UK. Sara Webb, Royal College of Midwives, UK. Sarah Hillman, Warwick Medical School, UK. Nicola J. Adderley, Department of Applied Health Sciences, School of Health Sciences, College of Medicine and Health, University of Birmingham, Edgbaston, Birmingham, UK; National Institute for Health and Care Research (NIHR) Birmingham Biomedical Research Centre, UK. Alice Sitch, Department of Applied Health Sciences, School of Health Sciences, College of Medicine and Health, University of Birmingham, Edgbaston, Birmingham, UK. Olalekan Lee Aiyegbusi, Department of Applied Health Sciences, School of Health Sciences, College of Medicine and Health, University of Birmingham, Edgbaston, Birmingham, UK. Marian Knight, National Perinatal Epidemiology Unit, University of Oxford, UK. Krishnarajah Nirantharakumar, Department of Applied Health Sciences, School of Health Sciences, College of Medicine and Health, University of Birmingham, Edgbaston, Birmingham, UK. Jane Whitehurst, NIHR Applied Research Collaboration West Midlands, UK.

## Supporting information


**Appendix S1.** Scoping review search terms.

**Appendix S2.** Inclusion and exclusion criteria categorized into population, concept, or context (PCC).

**Table S1.** Characteristics of included studies.

## Data Availability

The data that supports the findings of this study are available in the supplementary material of this article.
