# Supplementary material for: Healing‐assessment tools for perineal and cesarean section wounds in postpartum women: A scoping review
Source: Acta Obstet Gynecol Scand. 2025 Nov 11;105(1):18–29. doi: 10.1111/aogs.70089 (PMC12746225; doi:10.1111/aogs.70089)
Supplement: Supplementary file 1 — Appendix S1. Scoping review search terms. [file AOGS-105-18-s001.pdf]

S1: database search terms

Perineal trauma wound assessment tool searches

Ovid MEDLINE(R) and In-Process, In-Data-Review & Other Non-Indexed Citations

Search History (30)

#	Searches	Results
1	*Anal Canal/in	1049
2	exp Obstetric Labor Complications/ or exp Parturition/ or (birth or childbirth or labo?r).ab,kw,ti.	523531
3	1 and 2	578
4	(obstetric* and anal and sphincter and (tear* or injur* or trauma or lacerat* or wound* or rupture*)).ab,kw,ti.	1201
5	(OASIS or OASI).ab,kw,ti.	4326
6	((third or 3rd or 4th or fourth) and degree and (perineal or perineum or vagina*) and (tear* or injur* or trauma or lacerat* or wound* or rupture*)).ab,kw,ti.	948
7	*Perineum/in [Injuries]	1184
8	exp Perineum/	10592
9	exp "Wounds and Injuries"/	1029786
10	8 and 9	1446
11	((perineal or perineum or vagina*) and (tear* or injur* or trauma or lacerat* or wound* or rupture*)).ab,kw,ti.	17321
12	2 and (7 or 10 or 11)	7680
13	((childbirth or birth or labo?r or obstetric* or deliver*) and related and (perineal or perineum or vagina*) and (tear* or injur* or trauma or lacerat* or wound* or rupture*)).ab,kw,ti.	1075
14	exp Episiotomy/	2491
15	(episiotomy or episiotomies).ab,kw,ti.	3411
16	14 or 15	4057
17	4 or 5 or 6	5765
18	3 or 17 or 12 or 13 or 16	15022
19	exp Wound Healing/	146878
20	wound assessment tool.mp.	120
21	healing score.mp.	194
22	WAT.mp.	5493
23	wound assessment.mp.	736
24	wound healing.mp.	164251
25	wound healing questionnaire.mp.	10
26	healing tool.mp.	25
27	wound score.mp.	94
28	healing.mp.	284592
29	19 or 20 or 21 or 22 or 23 or 24 or 25 or 26 or 27 or 28	312623
30	18 and 29	622

Embase

# ▲	Searches	Results
1	exp anus injury/	994
2	(exp anus sphincter/ or exp anal canal/) and exp injury/	3831
3	exp Obstetric Labor Complications/ or exp Parturition/ or (birth or childbirth or labo?r or peri-partum or post-partum).ab,kw,ti.	772198
4	(1 or 2) and 3	1160
5	(obstetric* and anal and sphincter and (tear* or injur* or trauma or lacerat* or wound* or rupture*)).ab,kw,ti.	2171
6	(OASIS or OASI).ab,kw,ti.	6025
7	((third or 3rd or 4th or fourth) and degree and (perineal or perineum or vagina*) and (tear* or injur* or trauma or lacerat* or wound* or rupture*)).ab,kw,ti.	1764
8	exp perineum/ or exp perineum muscle/	14238
9	exp injury/	2702857
10	8 and 9	4118
11	((perineal or perineum or vagina*) and (tear* or injur* or trauma or lacerat* or wound* or rupture*)).ab,kw,ti.	29598
12	10 or 11	31142
13	((childbirth or birth or labo?r or deliver*) and related and (perineal or perineum or vagina*) and (tear* or injur* or trauma or lacerat* or wound* or rupture*)).ab,kw,ti.	1821
14	exp Episiotomy/	5813
15	(episiotomy or episiotomies).ab,kw,ti.	4765
16	4 or 5 or 6 or 7	8695
17	3 and 12	12710
18	13 or 14 or 15	8055
19	16 or 17 or 18	23518
20	limit 19 to "remove medline records"	9909
21	exp wound healing/	200534
22	exp wound assessment/	2578
23	wound assessment tool.mp.	165
24	healing score.mp.	279
25	WAT.mp.	7870
26	wound assessment.mp.	3121
27	wound healing.mp.	212302
28	wound healing questionnaire.mp.	17
29	healing tool.mp.	40
30	wound score.mp.	140
31	healing.mp. or exp healing/	464174
32	21 or 22 or 23 or 24 or 25 or 26 or 27 or 28 or 29 or 30 or 31	473513
33	20 and 32	401

Save Remove Combine with: AND OR

CINAHL via EBSCOhost

S34	S21 AND S33
S33	S22 OR S23 OR S24 OR S25 OR S26 OR S27 OR S28 OR S29 OR S30 OR S31 OR S32
S32	"healing"
S31	"wound score"
S30	"healing tool"
S29	"wound healing"
S28	"wound assessment"
S27	"WAT"
S26	"wound assessment tool"
S25	(MH "Clinical Assessment Tools+")
S24	"healing score"
S23	(MH "Wound Assessment+")
S22	(MH "Wound Healing+")
S21	S14 OR S15 OR S16 OR S17 OR S18 OR S19 OR S20
S20	TI (episiotomy or episiotomies) OR AB (episiotomy or episiotomies)
S19	(MM "Episiotomy")
S18	TI ((childbirth or birth or labo?r or deliver*) and related and (perineal or perineum) and (tear* or injur* or trauma or lacerat* or wound* or rupture*)) OR AB ((childbirth or birth or labo?r or deliver*) and related and (perineal or perineum) and (tear* or injur* or trauma or lacerat* or wound* or rupture*))
S17	AB ((third or 3rd or 4th or fourth) and degree and (perineal or perineum) and (tear* or injur* or trauma or lacerat* or wound* or rupture*)) OR TI ((third or 3rd or

	4th or fourth) and degree and (perineal or perineum) and (tear* or injur* or trauma or lacerat* or wound* or rupture*))
S16	TI (OASIS or OASI) OR AB (OASIS OR OASI)
S15	TI (obstetric* and anal and sphincter and (tear* or injur* or trauma or lacerat* or wound* or rupture*)) OR AB (obstetric* and anal and sphincter and (tear* or injur* or trauma or lacerat* or wound* or rupture*))
S14	S8 AND S13
S13	S9 OR S10 OR S11 OR S12
S12	TI (birth or childbirth or labo#r) OR AB (birth or childbirth or labo#r)
S11	(MH "Childbirth+")
S10	(MH "Delivery, Obstetric+")
S9	(MH "Labor Complications+")
S8	S1 OR S2 OR S7
S7	S5 AND S6
S6	S3 OR S4
S5	(MH "Wounds and Injuries+")
S4	(MM "Anus")
S3	(MM "Perineum")
S2	(MH "Anus/IN")
S1	(MH "Perineum/IN") OR TI((perineal or perineum) and ((tear* or injur* or trauma or lacerat* or wound* or rupture*)) OR AB ((perineal or perineum) and (tear* or injur* or trauma or lacerat* or wound* or rupture*))

Caesarean section wound assessment tool searches

Medline Ovid MEDLINE(R) and In-Process, In-Data-Review & Other Non-Indexed Citations

Search History (16)

#	Searches	Results	Type	Actions	
1	exp Wound Healing/	147064	Advanced	Display Results	More
2	wound assessment tool.mp.	120	Advanced	Display Results	More
3	healing score.mp.	197	Advanced	Display Results	More
4	WAT.mp.	5507	Advanced	Display Results	More
5	wound assessment.mp.	739	Advanced	Display Results	More
6	wound healing.mp.	164674	Advanced	Display Results	More
7	wound healing questionnaire.mp.	10	Advanced	Display Results	More
8	healing tool.mp.	25	Advanced	Display Results	More
9	wound score.mp.	94	Advanced	Display Results	More
10	healing.mp.	285352	Advanced	Display Results	More
11	1 or 2 or 3 or 4 or 5 or 6 or 7 or 8 or 9 or 10	313421	Advanced	Display Results	More
12	caesarean section.mp. or exp Cesarean Section/	63016	Advanced	Display Results	More
13	caesarean.mp.	25585	Advanced	Display Results	More
14	csection.mp.	4	Advanced	Display Results	More
15	12 or 13 or 14	66038	Advanced	Display Results	More
16	11 and 15	2474	Advanced	Display Results	More

Embase

Search History (18)

#	Searches	Results	Type	Actions	
1	exp wound healing/	201138	Advanced	Display Results	More
2	exp wound assessment/	2590	Advanced	Display Results	More
3	wound assessment tool.mp.	166	Advanced	Display Results	More
4	healing score.mp.	280	Advanced	Display Results	More
5	WAT.mp.	7886	Advanced	Display Results	More
6	wound assessment.mp.	3133	Advanced	Display Results	More
7	wound healing.mp.	213043	Advanced	Display Results	More
8	wound healing questionnaire.mp.	17	Advanced	Display Results	More
9	healing tool.mp.	40	Advanced	Display Results	More
10	wound score.mp.	140	Advanced	Display Results	More
11	healing.mp. or exp healing/	465529	Advanced	Display Results	More
12	1 or 2 or 3 or 4 or 5 or 6 or 7 or 8 or 9 or 10 or 11	474888	Advanced	Display Results	More
13	caesarean section.mp. or exp cesarean section/	134799	Advanced	Display Results	More
14	csection.mp.	144	Advanced	Display Results	More
15	caesarean.mp.	42702	Advanced	Display Results	More
16	13 or 14 or 15	137752	Advanced	Display Results	More
17	12 and 16	1734	Advanced	Display Results	More
18	limit 17 to "remove medline records"	831	Advanced	Display Results	More

Save Remove Combine with: AND OR

CINAHL

S8	S1 AND S6 **Limiters** - Exclude MEDLINE records; Human
S7	S1 AND S6
S6	S2 OR S3 OR S4 OR S5
S5	AB csection
S4	AB cesarean
S3	AB "caesarean"
S2	(MH "Cesarean Section+") OR "caesarean section"
S1	(MH "Wound Healing+") OR (MH "Wound Assessment+") OR "healing score" OR (MH "Clinical Assessment Tools+") OR "wound assessment tool" OR "WAT" OR "wound assessment" OR "wound healing" OR "healing tool" OR "wound score" OR "healing"

Google scholar- first 200 results reviewed (sorted by relevance)

("perineal trauma" OR "perineal tear" OR "childbirth tear" OR "perineal laceration" OR "episiotomy" OR "second degree tear" OR "first degree tear" OR "third degree tear" OR "fourth degree tear" OR "1st degree tear" OR "2nd degree tear" OR "3rd degree tear" OR "fourth degree tear" OR "OASIS" OR "OASI" OR "anal sphincter injury") AND ("wound healing" OR "healing" OR "wound assessment" OR "wound assessment tool" OR "wound tool")
