# Supplementary material for: Healing‐assessment tools for perineal and cesarean section wounds in postpartum women: A scoping review
Source: Acta Obstet Gynecol Scand. 2025 Nov 11;105(1):18–29. doi: 10.1111/aogs.70089 (PMC12746225; doi:10.1111/aogs.70089)
Supplement: Supplementary file 2 — Appendix S2. Inclusion and exclusion criteria categorized into population, concept, or context (PCC). [file AOGS-105-18-s002.docx]

**Appendix S2**

Categorisation of inclusion and exclusion criteria according to the Population, Concept, Context (PCC) framework

| **Inclusion criteria** | |
| --- | --- |
| Population | - Women of any age or cultural background who have childbirth-related perineal trauma or a caesarean section wound   - Childbirth-related perineal trauma of any type   - Sustained at assisted/spontaneous birth |
| Concept | - Wound assessment tool used to assess wound healing   - This was identified if the study utilised a range of symptoms or signs to determine an overall score, which established how well the wound was healing, or if a known tool was used |
| Context | - Any geographical location - Any timepoint in the postpartum period - No limits placed on year of publication - Primary research study utilising the tool |
| **Exclusion criteria** | |
| Population | - Patients with non-birth related wounds |
| Concept | - Tool used to assess scar cosmesis and not wound healing - Tool used to assess degree of perineal trauma initially sustained and not wound healing - Tool used purely for assessment on presence/absence of wound healing rather than for global wound healing assessment |
| Context | - Non-English language study - Publication not primary research - Tool development study not application of the tool |
