# Supplementary material for: Healing‐assessment tools for perineal and cesarean section wounds in postpartum women: A scoping review
Source: Acta Obstet Gynecol Scand. 2025 Nov 11;105(1):18–29. doi: 10.1111/aogs.70089 (PMC12746225; doi:10.1111/aogs.70089)
Supplement: Supplementary file 3 — Table S1. Characteristics of included studies. [file AOGS-105-18-s003.docx]

**Table S1: study characteristics**

| **Author (year)** | **Study location, study design, numbers of patients** | **Aim of study** | **Type of wound** | **Time evaluated (days postpartum)** | **Wound healing score name** | **Parameters** |
| --- | --- | --- | --- | --- | --- | --- |
| Abbasinia 2015 (abstract) | Unclear, randomised controlled trial, n=120 | To determine the effect of perforan ointment on episiotomy wound healing | Perineal trauma: episiotomy | 5th and 10th days | REEDA | Unclear |
| Abdollahpour 2023 | Iran, randomised controlled trial, n=74 | To evaluate the effect of Lactobacillus casei oral supplementation on episiotomy wound healing among primiparous women. | Perineal trauma: episiotomy | Before discharge, 5+/- 1 day and 15+/-1 postpartum | REEDA | Redness, oedema, ecchymosis, drainage/discharge, approximation |
| Ahmed 2022 | Egypt, randomised controlled trial, n=100 | To test the effect of a designed protocol of care on the outcomes of postpartum perineal trauma | Perineal trauma: 1st or 2nd degree tear or episiotomy | Initial assessment (pre-intervention), 6 hours, 7th day | REEDA | Redness, oedema, ecchymosis, drainage/discharge, approximation |
| Alvarenga 2017 | Brazil, randomised controlled trial, n=54 | To assess the effect of low level laser therapy on pain and perineal healing after an episiotomy | Perineal trauma: episiotomy | 6-10 hours, 20-24 hours, 40-48 hours, 7-10 days postpartum | REEDA | Redness, oedema, ecchymosis, drainage/discharge, approximation |
| Asgharikhatooni 2015 | Iran, randomised controlled trial, n=108 | To examine the effectiveness of topical application of Equisetum arvense ointment in wound healing, reduction of inflammation and pain relief after episiotomy in nulliparous mothers. | Perineal trauma: episiotomy | Baseline, 5+/1 day, 10 +/1 day postpartum | REEDA | Redness, oedema, ecchymosis, drainage/discharge, approximation |
| AydinBesen 2020 | Turkey, randomised controlled trial, n=56 | To determine the effect of continuous technique for repair of episiotomy and spontaneous tear repair compared to interrupted suture technique for perineal pain and dyspareunia | Perineal trauma: episiotomy and 2nd degree tear | 24 hours postpartum | REEDA | Redness, oedema, ecchymosis, drainage/discharge, approximation |
| Brezeanu 2022 | Romania, randomised controlled trial, n=20 | To assess the effect of lactic acid and collagen containing gel on episiotomy wound healing | Perineal trauma: episiotomy | 7th and 40th days | REEDA | Unclear |
| Caroci-Becker 2021 | Brazil, case series, n=19 | To describe the use of surgical glue to repair perineal trauma during normal delivery | Perineal trauma: 1st or 2nd degree tear or episiotomy | 2 hours, 12-24 hours and 36-48 postpartum | REEDA | Redness, oedema, ecchymosis, drainage/discharge, approximation. |
| Caroci-Becker 2023 | Brazil, randomised controlled trial, n=140 | To evaluate the effectiveness of n-butyl-2-cyanoacrylate surgical glue compared to the polyglactin 910 suture in repairing first- and second-degree perineal tears and episiotomy in vaginal births. | Perineal trauma: 1st or 2nd degree tear or episiotomy | 2h, 12-24h, 36-48h, 10-20 days, 50-70 days, 6-8 months postpartum | REEDA | Redness, oedema, ecchymosis, drainage/discharge, approximation. |
| Cheshfar 2023 | Iran, randomised controlled trial, n=70 | To investigate ginger extract ointment’s effects on the pain and recovery of episiotomy incisions in nulliparous women. | Perineal trauma: episiotomy | Baseline, 5+/1 day, 10 +/1 day postpartum | REEDA | Redness, oedema, ecchymosis, drainage/discharge, approximation. |
| Chougala 2015 | India, randomised controlled trial, n=60 | To evaluate the effect of therapeutic ultrasound and low-level laser therapy on perineal pain following vaginal delivery with episiotomy | Perineal trauma: episiotomy | 1st and 3rd days | REEDA | Redness, oedema, ecchymosis, drainage/discharge, approximation. |
| Cobanoglu 2020 | Turkey, randomised controlled trial, n=135 | To investigate whether the use of Hypericum perforatum oil used for wound care for women who had received an episiotomy to facilitate labour could be effective in facilitating healing. | Perineal trauma: episiotomy | 4 hours, 5th and 10th day postpartum | REEDA | Redness, oedema, ecchymosis, drainage/discharge, approximation. |
| David 1976 | Israel, randomised controlled trial, n=100 | To determine whether use of oxyphenbutazone reduces inflammation at the episiotomy site | Perineal trauma: episiotomy | 1st, 3rd, 5th days | Other | Oedema, hyperemia, pain |
| DeAngelis 2022 | Italy, randomised controlled trial, n=100 | To evaluate use of calendula ointment on pain after episiotomy in women with singleton pregnancy, and spontaneous labour at term | Perineal trauma: episiotomy | Each day from 1st to 10th days postpartum | REEDA | Redness, oedema, ecchymosis, drainage/discharge, approximation. |
| Dehghanpour 2023 | Iran, non-randomised clinical study, n=80 | To evaluate photobiomodulation on caesarean section wound healing | Caesarean section wound | 1st ,3rd ,7th ,10th days postpartum | REEDA | Redness, oedema, ecchymosis, drainage/discharge, approximation. |
| Dorbati 2018 | Iran, randomised controlled trial, n=128 | To compare the effects of Alpha® Ointment in comparison with Betadine® solution on episiotomy healing process. | Perineal trauma: episiotomy | 1st ,3rd ,7th days | REEDA | Redness, oedema, ecchymosis, drainage/discharge, approximation. |
| Eghdampour 2013 | Iran, randomised controlled trial, n=111 | To determine the impact of Aloe vera and Calendula on episiotomy healing in primiparous women. | Perineal trauma: episiotomy | 5th day | REEDA | Redness, oedema, ecchymosis, drainage/discharge, approximation. |
| Elkhouly 2021 | Egypt, randomised controlled trial, n=200 | To evaluate the effect of autologous platelet rich plasma on wound healing and pain perception after caesarean section | Caesarean section wound | 1st, 7th days and 6 months | REEDA | Redness, oedema, ecchymosis, drainage/discharge, approximation. |
| Ernawati 2020 | Indonesia, randomised controlled trial, n=30 | To evaluate post-caesarean section wound healing on patient given standard analgesia compared with tranversus abdominis plane block and wound site infiltration based on REEDA scale and platelet Lymphocyte Ratio 72 hours post-caesarean section | Caesarean section wound | 3rd day | REEDA | Redness, oedema, ecchymosis, drainage/discharge, approximation. |
| FaalSiahkal 2023 | Iran, randomised controlled trial, n=300 | To compare the continuous non-locking technique with interrupted suturing for the repair of episiotomy or second-degree perineal tears | Perineal trauma: episiotomy and 2nd degree tear | 1st, 10th, 42nd days | REEDA | Redness, oedema, ecchymosis, drainage/discharge, approximation. |
| Faraji 2021 | Iran, randomised controlled trial, n=90 | To investigate the efficacy and safety of myrrh- and frankincense-based sitz-baths on episiotomy wound healing in primiparous women. | Perineal trauma: episiotomy | 2nd and 7th days | REEDA | Redness, oedema, ecchymosis, drainage/discharge, approximation. |
| Fleming 2003 | UK, randomised controlled trial, n=1314 | To examine differences in outcome between primiparous women who do and who do not have suturing to first or second degree perineal lacerations sustained during spontaneous vaginal births after 37 weeks of gestation | Perineal trauma: 1st or 2nd degree tear | 1st, 10th and 42nd days | REEDA | Redness, oedema, ecchymosis, drainage/discharge, approximation. |
| Gaikwad 2019 | India, quasi-experimental study, n=60 | To assess the condition of episiotomy wound and assess the effect of pineapple extract on episiotomy wound healing. | Perineal trauma: episiotomy | Unclear | REEDA | Redness, oedema, ecchymosis, drainage/discharge, approximation. |
| Ghana 2017 | Iran, randomised controlled trial, n=178 | To investigate the effects of an abdominal binder on wound healing and consumed pain medication. | Caesarean section wound | 5th day | REEDA | Redness, oedema, ecchymosis, drainage/discharge, approximation. |
| Golezar 2016 | Iran, randomised controlled trial, n=82 | To determine the effect of oral bromelain on perineal pain and wound healing after episiotomy in primiparous women. | Perineal trauma: episiotomy | 3rd, 7th, 14th days | REEDA | Redness, oedema, ecchymosis, drainage/discharge, approximation. |
| Greer 1984 | UK, randomised controlled trial, n=40 | To compare the effects of pramoxine with hydrocortisone in relieving episiotomy discomfort and in improving episiotomy healing | Perineal trauma: episiotomy | 1st, 3rd, 5th | Other | Oedema, erythema, pain, impairment of mobility, analgesia requirement |
| Gul 2023 | Turkey, retrospective cohort, n=450 | To determine the effects of boron-based gel on episiotomy wound healing and episiotomy pain in primiparous women. | Perineal trauma: episiotomy | 2nd, 5-7th and 10-14th days | REEDA | Redness, oedema, ecchymosis, drainage/discharge, approximation. |
| Hadizadeh-Talasaz 2022 | Iran, randomised controlled trial, n=80 | To determine the effect of rosemary cream on episiotomy wound healing in primiparous women | Perineal trauma: episiotomy | Before intervention, 4th and 10th days | REEDA | Redness, oedema, ecchymosis, drainage/discharge, approximation. |
| Hajhashemi 2018 | Iran, randomised controlled trial, n=140 | To assess the efficacy of Achillea millefolium and Hypericum perforatum ointments on episiotomy wound healing in primiparous women. | Perineal trauma: episiotomy | 7th, 10th, 14th days | Modified REEDA | Redness, edema, ecchymosis, wound dehiscence and wound secretion |
| Harpreet 2016 | India, quasi-experimental study, n=60 | To assess the effectiveness of lavender oil on healing of episiotomy wound to provide maximum comfort to postpartum women and its comparison with povidine – iodine. | Perineal trauma: episiotomy | 1st, 3rd and 5th days | REEDA | Redness, oedema, ecchymosis, drainage/discharge, approximation. |
| Heidari 2013 | Iran, randomised controlled trial, n=130 | To investigate the effect of Iranian Astragalus gossypinus honey on healing casesarean operation wounds and scars | Caesarean section wound | 10th and 40th day postpartum | REEDA | Redness, oedema, ecchymosis, drainage/discharge, approximation. |
| Hemalatha Devi 2022 | India, randomised controlled trial, n=96 | The assessment of perineal pain post-episiotomy repair, with Trusynth Fast® versus Vicryl Rapide® suture. | Perineal trauma: episiotomy | 2nd, 11th, 42nd days | REEDA | Redness, oedema, ecchymosis, drainage/discharge, approximation. |
| Hill 1989 | USA, randomised controlled trial, n=90 | To evaluate the effects of cold and heat to the perineum | Perineal trauma: all types | Before or 2 hours after treatment | REEDA | Redness, oedema, ecchymosis, drainage/discharge, approximation. |
| Izadpanah 2019 | Iran, randomised controlled trial, n=129 | To determine the effect of grape seed extract ointment on CS wound healing | Caesarean section wound | before intervention, 6th and 14th day postpartum | REEDA | Redness, oedema, ecchymosis, drainage/discharge, approximation. |
| Kamble 2019 | India, quasi-experimental study, n=40 | To assess the effectiveness of Povidone Iodine Ointment versus Framycetin Sulphate Cream over episiotomy wound healing among postnatal mothers. | Perineal trauma: episiotomy | 1st, 2nd, 3rd, 4th, 5th days | Modified REEDA | Redness, oedema, ecchymosis, drainage/discharge, approximation. |
| Kanwar 2018 | India, quasi-experimental study, n=60 | To evaluate the effectiveness of medicated versus non medicated sitz bath in episiotomy healing among postnatal mothers of selected areas of Mohali, Punjab | Perineal trauma: episiotomy | 3rd, 5th , 7th days | REEDA | Redness, oedema, ecchymosis, drainage/discharge, approximation. |
| Kaur 2015 | India, quasi-experimental study, n=60 | To assess the effectiveness of infra-red therapy upon level of episiotomy pain and wound healing | Perineal trauma: episiotomy | 1st, 2nd, 3rd days | Modified REEDA | Redness, oedema, ecchymosis, drainage/discharge, approximation. |
| Kaviani 2017 | Iran, randomised controlled trial, n=90 | This study aimed to assess the impact of olive leaf extract ointment on pain intensity and early maternal complications in primiparous women. | Perineal trauma: episiotomy | 1st, 3rd, 7th, 10th, 14th | REEDA | Redness, oedema, ecchymosis, drainage/discharge, approximation. |
| Kazemi 2021 | Iran, randomised controlled trial, n=130 | To determine the effect of green tea ointment on pain and wound healing after episiotomy. | Perineal trauma: episiotomy | 5th and 10th days | REEDA | Redness, oedema, ecchymosis, drainage/discharge, approximation. |
| Khade 2019 | India, quasi-experimental study, n=80 | To assess the effectiveness of honey application on LSCS wound healing. | Caesarean section wound | 1st and 6th days | REEDA | Not clear |
| Khadivzadeh 2009 (abstract) | Iran, randomised controlled trial, n=90 | To compare the perineal pain and wound healing in primiparous women in two groups of women who were administered lavender cream or placebo | Perineal trauma: episiotomy | 1st, 3rd, 10th days | REEDA | Not clear |
| Khodabakhshi 2013 | Iran, quasi-experimental study, n=60 | To determine the effect of onion on healing of episiotomies | Perineal trauma: episiotomy | Unclear | REEDA | Redness, oedema, ecchymosis, drainage/discharge, approximation. |
| Kilic 2021 | Turkey, randomised controlled trial, n=869 | To compare wound dressing removal at 24 hours versus 48 hours following low-risk caesarean deliveries in terms of wound healing | Caesarean section wound | 7th and 42nd days | ASEPSIS | Additional treatment (drainage/antibiotics/debridement), serous discharge, erythema, purulent exudate, separation of deep tissues, isolation of bacteria, stay as inpatient prolonged over 14 days. |
| Kindberg 2008 | Denmark, randomised controlled trial, n=400 | To compare a continuous suture technique with interrupted stitches using inverted knots for postpartum perineal repair of second-degree lacerations and episiotomies. | Perineal trauma: episiotomy and 2nd degree tear | 1st-2nd and 10th days | REEDA | Redness, oedema, ecchymosis, drainage/discharge, approximation. |
| Lallemant 2023 | France, randomised controlled trial, n=143 | To assess the preliminary efficacy and safety of conservative management compared with systematic suture in isolated vaginal or first-degree perineal tears after birth | Perineal trauma: 1st degree | 3rd, 10th days, 2 months postpartum | REEDA | Redness, oedema, ecchymosis, drainage/discharge, approximation. |
| Lavaf 2018 | Iran, randomised controlled trial, n=120 | To investigate and compare the effect of phenytoin and honey cream on intensity of pain and episiotomy wound healing in nulliparous women | Perineal trauma: episiotomy | Within 1st day, 7th and 14th days | REEDA | Redness, oedema, ecchymosis, drainage/discharge, approximation. |
| Limbachiya 2022 | India, quasi-experimental study, n=60 | To determine the association between Episiotomy pain level and wound healing among postpartum women with their demographic variable. | Perineal trauma: episiotomy | 1st, 2nd, 3rd days | REEDA | Redness, oedema, ecchymosis, drainage/discharge, approximation. |
| Lopez-Lapeyrere 2020 | Spain, randomised controlled trial, n=168 | To compare three perineal repair suturing techniques after episiotomy or second degree tearing during a normal birth with respect to reducing pain and improving the perineal healing process. | Perineal trauma: episiotomy and 2nd degree tear | 2 hours, discharge, 10th day | REEDA | Redness, oedema, ecchymosis, drainage/discharge, approximation |
| Mahmudi 2015 | Iran, randomised trial, n=181 | To investigate the effect of turmeric cream on caesarean section wound healing | Caesarean section wound | 1st, 7th and 14th days | REEDA | Redness, oedema, ecchymosis, drainage/discharge, approximation |
| Majid 2023 | Pakistan, prospective cohort, n=70 | To compare the effects of honey and povidone-iodine combination with povidone-iodine alone, to determine wound healing after caesarean section | Caesarean section wound | 5th, 7th, 9th and 10th days | ASEPSIS | Additional treatment (drainage/antibiotics/debridement), serous discharge, erythema, purulent exudate, eparation of deep tissues, isolation of bacteria, stay as inpatient prolonged over 14 days. |
| Manjula 2012 | India, prospective cohort, n=60 | To examine the factors influencing episiotomy wound healing | Perineal trauma: episiotomy | Unclear | REEDA | Redness, oedema, ecchymosis, discharge, approximation |
| Marks 2020 | Brazil, cross sectional (aligned with randomised controlled trial) | To compare the intensity of perineal pain, the healing process and satisfaction postpartum of women who sustained first and second degree tears/episiotomy after childbirth and underwent repair with glue or fast absorbing Vicryl suture | Perineal trauma: 1st or 2nd degree tear or episiotomy | Between days 10-20 | REEDA | Redness, oedema, ecchymosis, drainage/discharge, approximation |
| Meng 2022 | China, randomised controlled trial, n=700 | To evaluate the effect of alginate and gelatin hydrogel composited with nano-sinc on caesarean section wound healing | Caesarean section wound | 2nd, 7th and 14th days | REEDA | Redness, oedema, ecchymosis, drainage/discharge, approximation |
| Mohaghegh 2022 | Iran, randomised controlled trial, n=60 | To assess the effect of Malva Sylvestris cream on episiotomy pain and healing | Perineal trauma: episiotomy | 1st, 7th and 14th days | REEDA | Redness, oedema, ecchymosis, drainage/discharge, approximation |
| Mohammed Pour 2020 | Iran, randomised controlled trial, n=86 | To investigate the effect of Eremostachy laciniata on caesarean section pain and distress | Caesarean section wound | 5th day | REEDA | Redness, oedema, ecchymosis, drainage/discharge, approximation |
| Mohammadi 2014 check timings wounds checked | Iran, randomised controlled trial, n=144 | To assess the effect of cinnamon on perineal pain and healing of episiotomy | Perineal trauma: episiotomy | Before intervention, 8 hours after intervention and on 10th-11th day postpartum | REEDA | Redness, oedema, ecchymosis, drainage/discharge, approximation |
| Molazem 2014 | Iran, randomised controlled trial, n=90 | To determine the effectiveness of dressing caesarean section wounds with aloe vera gel to improve healing | Caesarean section wound | 1st and 8th day | REEDA | Redness, oedema, ecchymosis, drainage/discharge, approximation |
| Moudi 2018 | Iran, randomised controlled trial, n=147 | To investigate the effects of mastic oleoresin on wound healing and episiotomy pain intensity | Perineal trauma: episiotomy | 3rd, 7th, 10th day | REEDA | Redness, oedema, ecchymosis, drainage/discharge, approximation |
| Naviba 2009 | Iran, randomised controlled trial, n=121 | To investigate the effects of localised cooling treatment to alleviate perineal trauma | Perineal trauma: episiotomy | 4 hours post-episiotomy, 1st, 2nd, 5th and 10th days | REEDA | Redness, oedema, ecchymosis, drainage/discharge, approximation |
| Nikpour 2014 | Iran, randomised controlled trial, n=75 | To investigate whether honey can improve wound healing in caesarean section wounds | Caesarean section wound | 7th and 14th days | REEDA | Redness, oedema, ecchymosis, drainage/discharge, approximation |
| NK 2023 | India, randomised controlled trial, n=99 | To determine perineal pain and maternal morbidity after repair with Truglyde Fast or Safil Quick sutures | Perineal trauma: episiotomy | 2nd, 11th and 42nd day | REEDA | Redness, oedema, ecchymosis, drainage/discharge, approximation |
| Pakniat 2017 | Iran, randomised controlled trial, n=130 | To determine the effectiveness of phenytoin cream on wound healing after episiotomy | Perineal trauma: episiotomy | 1st, 5th and 10th day | REEDA | Redness, oedema, ecchymosis, drainage/discharge, approximation |
| Parimala 2020 | India, randomised controlled trial, n=100 | To determine the effectiveness of Betadine wash used to improve wound healing after episiotomy | Perineal trauma: episiotomy | Unclear | REEDA | Parameters not documented |
| Patel 2023 | India, quasi-experimental, n=40 | To evaluate the effect of topical breast milk application on postpartum women's episiotomy wound healing | Perineal trauma: episiotomy | 1st, 3rd, 5th and 7th day | REEDA | Redness, oedema, ecchymosis, drainage/discharge, approximation |
| Peralta 2020 (abstract | Phillipines, unclear, n=105 | To determine the effectiveness of using chlorhexidine impregnated braided absorbable suture and plain natural absorbable suture in preventing episiotomy repair dehiscence | Perineal trauma: episiotomy | 1 hour post-partum and 1st and 7th days | REEDA | Redness, oedema, ecchymosis, drainage/discharge, approximation |
| Rahmani 2023 | Iran, randomised controlled trial, n=100 | To determine the effect of Achillea vermicularis on wound healing after episiotomy | Perineal trauma: episiotomy | 7th and 10th days | REEDA | Redness, oedema, ecchymosis, drainage/discharge, approximation |
| Resmy 2019 | India, non-randomised controlled trial, n=60 | To determine the effectiveness of salt water application on episiotomy wound healing amongst postnatal mothers | Perineal trauma: episiotomy | Unclear | REEDA | Unclear |
| Rohmatin 2022 | Indonesia, quasi-experimental, n=28 | To determine the effect of honey on the duration of wound healing amongst postpartum mothers who underwent caesarean section | Caesarean section wound | Days 1-7 postpartum | REEDA | Redness, oedema, ecchymosis, drainage/discharge, approximation |
| Roma 2023 | Egypt, quasi-experimental, n=100 | To determine the effect of dry heat application on perineal pain and episiotomy wound healing | Perineal trauma: episiotomy | 5th and 10th days | REEDA | Redness, oedema, ecchymosis, drainage/discharge, approximation |
| Sahoo 2013 | India, quasi-experimental, n=40 | To determine the level of pain and wound healing in postnatal mothers after receiving infra-red therapy | Perineal trauma: episiotomy | 1st, 3rd and 7th day | REEDA | Redness, oedema, ecchymosis, drainage/discharge, approximation |
| Salvi 2022 | India, randomised controlled trial, n=100 | To compare the traditional suturing technique versus the continuous non-locking technique for the repair of episiotomy | Perineal trauma: episiotomy | 1st, 2nd, 3rd day | REEDA | Redness, oedema, ecchymosis, drainage/discharge, approximation |
| Salvi 2022a | India, randomised controlled trial, n=100 | To evaluate the effectiveness of cold compression therapy versus infrared radiation therapy for healing of episiotomy wound | Perineal trauma: episiotomy | 1st and 3rd days | REEDA | Redness, oedema, ecchymosis, drainage/discharge, approximation |
| Samadi 2010 | Iran, randomised controlled trial, n=144 | To determine the effects of Hypericum perforatum on caesarean section wound healing | Caesarean section wound | 10th day | REEDA | Redness, oedema, ecchymosis, drainage/discharge, approximation |
| Santos 2012 | Brazil, randomised controlled trial, n=52 | To evaluate the effects of low-level laser therapy for perineal pain and healing after episiotomy | Perineal trauma: episiotomy | 2 hours postpartum, 1st, 2nd, 15th and 20th days | REEDA | Redness, oedema, ecchymosis, drainage/discharge, approximation |
| Sarbaz 2019 | Iran, randomised controlled trial, n=60 | To investigate the effect of sitz bath of hydro-alcohol extract of myrrh plant on episiotomy wound healing | Perineal trauma: episiotomy | 3rd, 7th, 10th day | REEDA | Redness, oedema, ecchymosis, drainage/discharge, approximation |
| Shahrahmani 2018a | Iran, randomised controlled trial, n=60 | To determine the effect of green tea ointment on the healing process of episiotomy | Perineal trauma: episiotomy | 1st, 5th, 10th days | REEDA | Redness, oedema, ecchymosis, drainage/discharge, approximation |
| Shahrahmani 2017 | Iran, randomised controlled trial, n=99 | To determine the effect of green tea ointment on episiotomy pain and wound healing | Perineal trauma: episiotomy | 1st, 5th, 10th days | REEDA | Redness, oedema, ecchymosis, drainage/discharge, approximation |
| Shayan 2020 | Iran, randomised controlled trial, n=80 | To investigate the effects of honey-olive oil combination on episiotomy wound healing and pain relief | Perineal trauma: episiotomy | 7th, 10th, 14th days | REEDA | Redness, oedema, ecchymosis, drainage/discharge, approximation |
| Sheikhan 2012 | Iran, randomised controlled trial, n=60 | To determine if lavender oil essence is effective in reducing perineal discomfort following episiotomy | Perineal trauma: episiotomy | Pre-intervention, 5th day | REEDA | Redness, oedema, ecchymosis, drainage/discharge, approximation |
| Swenson 2019 | USA, randomised controlled trial, n=35 | To determine if there is any difference in postpartum perineal pain between 3 different methods of skin closure | Perineal trauma: 2nd degree | 42 days | REEDA | Redness, oedema, ecchymosis, drainage/discharge, approximation |
| Taheri 2022 | Iran, randomised controlled trial, n=103 | To determine the effect of olive cream on the severity of pain and healing of caesarean section wounds | Caesarean section wound | 2nd and 10th days | REEDA | Redness, oedema, ecchymosis, drainage/discharge, approximation |
| Taleb 2021 | Iran, randomised controlled trial, n=93 | To determine the effect of Verbascum Thapsus on episiotomy wound healing | Perineal trauma: episiotomy | In the 'first hours after delivery', 1st, 3rd, 10th days | REEDA | Redness, oedema, ecchymosis, drainage/discharge, approximation |
| Tara 2009 | Iran, quasi-randomised controlled trial, n=63 | To determine the effect of turmeric ointment on healing of episiotomy wounds | Perineal trauma: episiotomy | 7th, 10th, 14th days | REEDA | Redness, oedema, ecchymosis, drainage/discharge, approximation |
| Tay 1999 | Singapore, randomised controlled trial, n=100 | To investigate the use of local application of procaine spirit versus cleaning with water for the care of episiotomy wounds | Perineal trauma: episiotomy | 1st, 2nd, 3rd, 7th and 14th days | Modified REEDA | Redness, oedema, ecchymosis, drainage/discharge, approximation |
| Tehranian 2016 | Iran, randomised controlled trial, n=140 | To evaluate the use of autologous platelet rich plasma on wound healing in high-risk women undergoing caesarean sections | Caesarean section wound | 1st, 5th, 56th days | REEDA | Redness, oedema, ecchymosis, drainage/discharge, approximation |
| Toomari 2021 | Iran, randomised controlled trial, n=87 | To determine the effect of Silybum marianum on pain severity and healing of episiotomy wounds | Perineal trauma: episiotomy | 12 hours post-delivery, 5th and 10th days | REEDA | Redness, oedema, ecchymosis, drainage/discharge, approximation |
| Torkashvand 2021 | Iran, randomised controlled trial, n=73 | To determine the effect of Olea ointment on episiotomy wound healing amongst primiparous women | Perineal trauma: episiotomy | Prior to intervention', 2 and 24 hours after intervention and 5th and 10th days after delivery | REEDA | Redness, oedema, ecchymosis, drainage/discharge, approximation |
| Teixeira 2020 | Brazil, randomised controlled trial, n=20 | To determine the feasibility of conducting a randomised controlled trial on use of Epiglu tissue adhesive to repair first degree tears | Perineal trauma: 1st degree | 0-2 hours, 12-24 hours, 36-48 hours, 10-20 days | REEDA | Redness, oedema, ecchymosis, drainage/discharge, approximation |
| Venkadalakshmi 2010 | India, randomised controlled trial, n=60 | To determine levels of pain and wound healing using infrared therapy after episiotomy | Perineal trauma: episiotomy | 1st, 2nd, 3rd days | REEDA | Redness, oedema, ecchymosis, drainage/discharge, approximation |
| Waghmare 2018 | India, quasi-experimental controlled trial, n=50 | To determine the effects of Calendula oil application on caesarean section wound healing | Caesarean section wound | 4th, 7th, 9th days | REEDA | Not specified |
| Walker 2012 (abstract) | UK, pilot randomised controlled trial, n=47 | To determine the rate of wound healing between beaded prolene and dexon | Caesarean section wound | Unclear | REEDA | Not specified |
| Yekta 2020 | Iran, randomised controlled trial, n=80 | To investigate the effect of Recove ointment on wound healing in women susceptible to caesarean section wound infection | Caesarean section wound | 1st, 3rd, 5th and 10th days | REEDA | Not specified |
| Zarei 2020 | Iran, quasi-experimental controlled trial, n=72 | To determine the effect of a self-care training program on wound healing after caesarean section | Caesarean section wound | 1st and 9th day | REEDA | Redness, oedema, ecchymosis, drainage/discharge, approximation |
| Zhang 2021 | China, randomised controlled trial, n=800 | To determine whether wound healing after caesarean section can be improved with an alginate-aloe vera/zinc oxide nanoparticle film | Caesarean section wound | 1st, 11th, 25th days | REEDA | Redness, oedema, ecchymosis, drainage/discharge, approximation |
| Zibanejad 2020 | Iran, randomised controlled trial, n=160 | To evaluate the effect of Q. persica and L inermis ointment on episiotomy wound healing | Perineal trauma: episiotomy | 1st, 7th, 10th and 14th days | REEDA | Redness, oedema, ecchymosis, drainage/discharge, approximation |
